# Burned-Out Testicular Germ Cell Tumor Presenting as Retroperitoneal Lymphadenopathy in a Patient With Cryptorchidism: A Case Report & Review of Literature

**DOI:** 10.7759/cureus.26776

**Published:** 2022-07-12

**Authors:** Soroush Shahrokh, Michelle Hebert, Woondong Jeong, Shan Guo

**Affiliations:** 1 Medicine, Hospital Corporation of America (HCA) Houston Healthcare Kingwood/University of Houston College of Medicine, Houston, USA; 2 Pathology, Hospital Corporation of America (HCA) Houston Healthcare Kingwood/University of Houston College of Medicine, Houston, USA; 3 Hematology & Medical Oncology, Hospital Corporation of America (HCA) Houston Healthcare Kingwood/University of Houston College of Medicine, Houston, USA

**Keywords:** teratoma, embryonal carcinoma, retroperitoneal lymph node dissection, cryptorchidism, mixed germ cell tumor, testicular cancer, burned-out tumor, burned-out testicular tumor, testicular germ cell tumor

## Abstract

Testicular germ cell tumors are the most common malignancy in young and middle-aged men. Spontaneous primary testicular tumor regression, or testicular tumor burn-out, is a rare clinical phenomenon where extragonadal metastatic lesions are observed concurrently with the spontaneous regression of the primary testicular germ cell tumors. Here, we describe the case of a 36-year-old male who presented to our hospital with left-sided abdominal pain and testicular swelling and was found to have significant retroperitoneal lymphadenopathy on his abdominopelvic CT scan. His testicular ultrasound showed multiple echogenic calcifications through the right testicle consistent with microlithiasis. Biopsy of the retroperitoneal lesion revealed a mixed germ cell tumor of testicular origin composed of embryonal carcinoma and teratoma. The patient received four cycles of bleomycin, etoposide, and cisplatin, followed by retroperitoneal lymph node dissection (RPLND) and radical right testicular orchiectomy. Here, we report the second case of burned-out testicular tumor in a patient with ipsilateral cryptorchidism. Furthermore, we elucidate the etiology, clinical presentation, and diagnostic modalities in burned-out testicular germ cell tumors.

## Introduction

Spontaneous primary tumor regression, or tumor burn-out, refers to the clinical phenomenon where the metastatic disease is present without any evidence of the primary tumor [1]. The burn-out phenomenon has been reported in various neoplasms, including hepatobiliary carcinoma, melanoma, and breast adenocarcinoma [2-4]. However, the predominant malignancy associated with the burn-out phenomenon is testicular germ cell tumors (GCT) [1]. Here, we present the case of a 36-year-old male with right testicular cryptorchidism who presented with a large retroperitoneal mass revealed to be metastasis from a burned-out right testicular GCT.

## Case presentation

**Figure 1:**
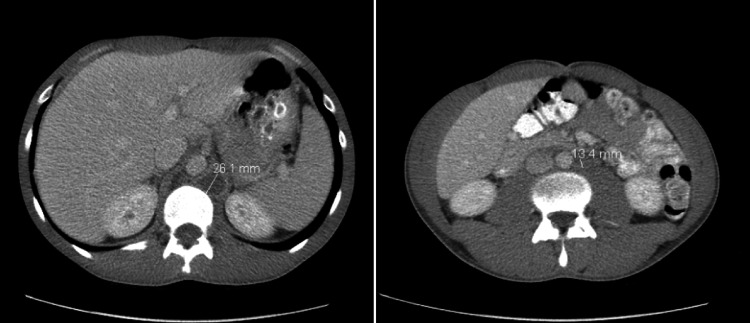
CT of the abdomen and pelvic showing large retroperitoneal lymphadenopathy, the largest conglomeration on the left side measuring 7.8 cm x 6.5 cm, encasing the left ureter causing moderate obstructive hydronephrosis.

**Figure 2:**
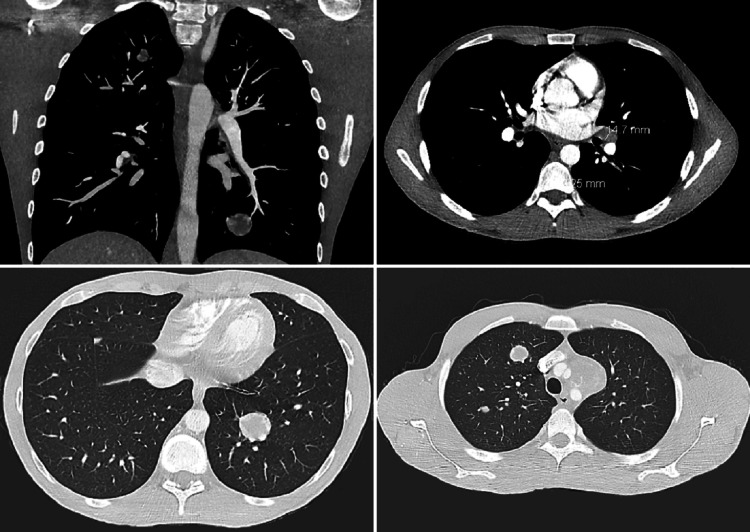
CT of the chest and mediastinum showing enlarged mediastinal lymph nodes, with the largest one measuring 3 cm, and multiple hilar lymph nodes measuring up to 1 cm.

**Figure 3:**
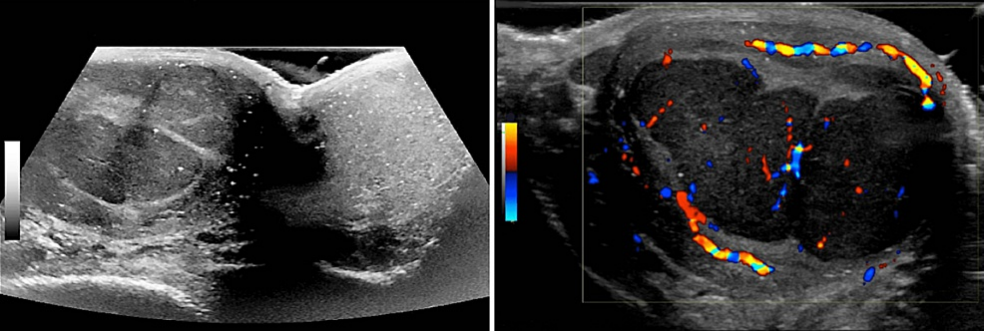
Testicular ultrasound showing multiple echogenic calcifications throughout the right testicle, the largest measuring 3 mm x 3 mm x 4 mm, consistent with testicular microlithiasis. There are no distinct testicular lesions suspected of malignancy.

A 36-year-old male with a history of right testicular cryptorchidism status post orchiopexy at the age of four presented to the hospital complaining of worsening left lower pelvic and groin pain for the past two months. He also had concurrent right testicular swelling, night sweats, and an unintentional 15 lbs weight loss. The patient’s physical exam revealed diffuse abdominal distention, mild left costovertebral angle tenderness, and a significantly enlarged right testicle. His abdominopelvic CT revealed diffuse retroperitoneal lymphadenopathy, the largest conglomeration measuring 7.8 cm x 6.5 cm, encasing the left ureter, causing moderate obstructive hydronephrosis (Figure 1). The chest CT revealed mediastinal and hilar lymph nodes (LN) enlargement measuring up to 3 cm (Figure 2), while testicular ultrasound revealed multiple right testicular echogenic microcalcifications, indicative of microlithiasis (Figure 3).

**Figure 4:**
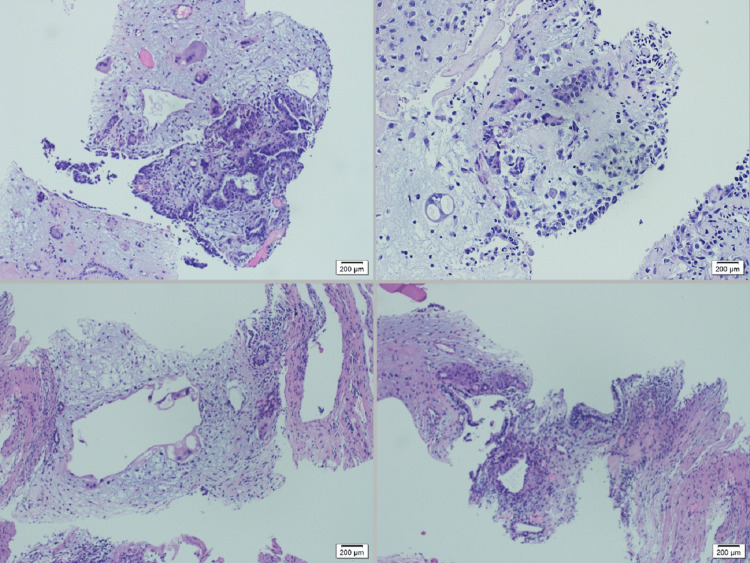
The microscopic exam of the left retroperitoneal lymph node demonstrating small fragments of neoplastic tissue admixed with skeletal muscle. The neoplastic tissue shows a myxoid and mucoid stroma with several multinucleated syncytiotrophoblasts consistent with embryonal carcinoma, while there are multiple benign glandular components consistent with a component of teratoma.

The patient’s serum tumor markers revealed a significantly elevated β-human chorionic gonadotropin, lactate dehydrogenase, and α-fetoprotein. He had a core needle biopsy of the left retroperitoneal lymph node, which showed small fragments of neoplastic tissue admixed with skeletal muscle, with myxoid stroma and multinucleated syncytiotrophoblasts consistent with embryonal carcinoma. Benign glandular elements were also present, suggesting a component of teratoma. The results were consistent with mixed GCT of testicular origin (Figure 4).

The patient’s positron emission tomography (PET) revealed increased metabolic activity in the mediastinal and retroperitoneal lymph nodes. He was diagnosed with stage 3C (cTxN3S2 M1a) mixed non-seminoma GCT. He received four cycles of neoadjuvant chemotherapy consisting of bleomycin 30 mg, etoposide 100 mg, and cisplatin 20 mg (BEP) once every three weeks for four cycles, without any treatment-related complications. The patient’s tumor markers were normalized post-chemotherapy, although his PET scan showed persistent para-aortic and retroperitoneal lymphadenopathy. The patient underwent retroperitoneal lymph-node dissection (RPLND), removing 14 para-aortic and paracaval lymph nodes, followed by radical right inguinal orchiectomy. The microscopic examination of paracaval lymph nodes revealed necrotic fibro-adipose tissue with hemorrhage but no neoplastic cells, and the dissected peri-aortic lymph nodes revealed one node containing mature teratoma while the remaining seven showed no neoplastic tissue. The histopathologic exam of the testicle revealed calcifications and scar tissue, confirming burned-out GCTs. The patient’s post-op PET scan showed no residual malignancy, and he has remained in remission until now, 11 months post-operation.

## Discussion

Burned-out testicular tumors are disseminated neoplastic lesions of testicular origin without an active malignancy in the testes [1]. Prym et al. first reported this in 1927 after finding disseminated choriocarcinoma in the post-mortem examination of a young man who had only fibrous scar tissue remnant in his right testicle with no viable neoplastic cells, which Prym attributed to the spontaneous regression of the primary testicular tumor [5]. Three decades later, Azzopardi et al. further characterized the phenomenon after finding unilateral testicular fibrous scar tissue remnants of undifferentiated neoplastic germ cells which had undergone a necrotic transformation in the post-mortem exam of 17 men who died from metastatic choriocarcinoma or embryonal cell carcinoma without a primary testicular tumor [6,7].

While the pathogenesis of spontaneous testicular tumor regression remains elusive, three leading theories have emerged: 1) T-cell mediated response against the primary testicular GCT occurs only after the extragonadal tumor dissemination, which allows for the cytotoxic T-cells to recognize and mount an immune response against the neoplastic cells; 2) primary testicular tumor ischemia caused by vascular supply and demand mismatch due to its high metabolic demands lead to primary tumor regression; 3) the de-novo development of neoplastic testicular germ cells occur in the extragonadal tissue [8-11]. In the case of our patient, spontaneous tumor regression is the most plausible scenario, as his testicular pathology showed foci of hyalinization and scarring without viable neoplastic cells, consistent with tumor regression.

A distinguishing feature of this case was the patient’s history of right testicle cryptorchidism, which was subsequently the primary tumor location. Although cryptorchidism is a well-known risk factor for the subsequent development of testicular cancer, to our best knowledge, only one prior study has reported a burned-out testicular tumor in a patient with cryptorchidism [12].

The diagnosis of burned-out testicular GCTs is challenging as it has no distinct clinical, laboratory, or imaging characteristics [12-14]. When burned-out testicular tumors are suspected, high-resolution ultrasound is the gold-standard imaging modality as it can detect small, highly echogenic foci of microcalcification and microlithiasis, a sonographic feature of burned-out tumors [13]. Interestingly, post-chemo imaging to detect residual malignancy is often associated with a high rate of false-positive findings. We have previously reported a similar case with post-chemo lymph node enhancement in a patient with burned-out testicular GCT, which we attributed to residual malignancy. However, subsequent RPLND revealed no viable tumor cells in the dissected lymph nodes, similar to the present case [14].

## Conclusions

Spontaneous primary tumor regression, or tumor burn-out, is a rare clinical phenomenon where there are metastatic tumor cells but with partial or complete disappearance of the primary tumor in the tissue of origin. Here, we reported a 36-year-old male with a history of ipsilateral right testicular cryptorchidism status post orchiopexy who presented with retroperitoneal lymphadenopathy diagnosed as a mixed germ-cell tumor of testicular origin composed of embryonal carcinoma and teratoma. However, his testicular ultrasound revealed no lesions suspicious of malignancy. The patient received four cycles of bleomycin, etoposide, and cisplatin, followed by radical right inguinal orchiectomy and RPLND. The microscopic examination of the right testicle revealed multiple microcalcifications and scar tissues confirming a burned-out testicular germ cell tumor. Meanwhile only one of the 14 dissected lymph nodes showed mature teratoma. The patient’s post-op imaging showed no residual malignancy, and he has remained in remission until now. This report describes the etiology, clinical presentation, and diagnostic modalities in burned-out testicular germ cell tumors. Furthermore, it examines the theories on the pathogenesis of testicular tumor burn-out. To our best knowledge, this is the second reported case of burned-out testicular tumor in a patient with ipsilateral cryptorchidism.
